# Art & anatomy: an everlasting relationship creating new insights in teaching surface anatomy

**DOI:** 10.15694/mep.2020.000023.1

**Published:** 2020-01-28

**Authors:** Vasileios Mitrousias, Kyriaki Baxevanidou, Aristeidis Zibis

**Affiliations:** 1 University Of Thessaly

**Keywords:** undergraduate medical education, anatomy, surface anatomy, art in teaching

## Abstract

This article was migrated. The article was marked as recommended.

The purpose of this study is to briefly present the unique relationship between art and anatomy and propose the use of art in teaching surface anatomy, evaluating its effectiveness through a randomized control study. The use of art paintings in teaching surface anatomy to undergraduate medical students was investigated. In the “Surface Anatomy” student selected component (SSC), art paintings instead of classic anatomical images were used as an intervention (art group, n=30; control group, n=15) during six hours of revision lectures. Perceptions of students and impact of art paintings on performance were investigated. The vast majority of students considered the use of art paintings as an interesting approach, which made lectures more interesting and improved understanding. No impact on performance was observed since mean examination scores did not differ significantly (Control group:73.9±9.4; Art group:78.8±8.6, p=0.10). Students also stated that the use of art paintings moderately improved their level of art knowledge and proposed a visit to a museum for a live anatomy lesson using paintings and sculptures. In conclusion, the use for art paintings in teaching and learning surface anatomy is highly appreciated by students, seems to improve understanding and makes the educational process more interesting. It should be furtherly investigated to be considered for inclusion in future curricula.

## Introduction

There is no better place to seek for the unique relationship between art and anatomy than the history of illustration. Illustration in anatomy dates back to 1500 B.C. when Egyptian, Babylonian, Chinese, and Indian civilizations provide us with the first medical illustrations on silk, metal or stone ( Calkins *et al*., 1999). Later in 350 B.C., Greeks tried to explain the structure and the function of the human body, and particularly Aristoteles, from Stagira (384-322 B.C.), who was the first who used a variety of paradigms, schemata and diagrams to teach his students ( O’Malley and Saunders, 1952).

However, it is not until the dawn of Renaissance that a prominent personality, not only for the art but also for the anatomy, makes its appearance. This is Leonardo da Vinci (1452-1519), who contributed to the progress in multiple sectors. During his life he made almost 30 dissections, thus understanding extensively the anatomy of the human body ( Netter, 1956). However, the key point in his work is the fact that Leonardo not only did the dissections on human bodies, but he also illustrated exactly what he saw ( O’Malley and Saunders, 1952). And except for the structure, Leonardo’s anatomical illustrations also provided data for the function of the human body ( Keele, 1979). Leonardo used his knowledge in order to teach other artists, especially Michelangelo and Raphael ( Sterpetti, Fiori and Ventura, 2017). In fact, he was the first to use art to teach anatomy to others and his collection of anatomy artwork is still popular today.

Michelangelo also studied dissected cadavers, although not mentioned as an anatomist by historians ( De Campos *et al.*, 2016). His interest in anatomy is reflected in his painting “the Last Judgement”, in which he depicted Saint Bartholomew to the left of Jesus, holding a flayed skin in his left hand and a knife in his right ( Eknoyan, 2000). It is also believed that he may have concealed symbols associated with female anatomy in the ceiling of the Sistine Chapel, in Rome ( De Campos *et al.*, 2016). Respectively, Raphael may have secretly depicted a human brain in his painting “The Transfiguration of Christ” ( Paluzzi *et al.*, 2007). In general, paintings of this period thought to have hidden meanings. Could these true masterpieces also hide an educational aspect?

It is for the first time so obvious, in this historical period, that anatomy and art are so closely related and interdependent. Artists need anatomy to draw their paintings. But anatomists also need artists to create proper illustrations. Indeed, later on, Estienne and Riviere, employed Rosso and Goujon, master artists of the Renaissance, for their textbook *De Dissectione Partium Corporis Humani.* Giovanni Battista Canano employed Girolamo da Carpi to illustrate his descriptions of muscles of the arm and even Andreas Vesalius (1514-1564) employed a pupil of the great painter Titian, Jan Stephan Calcar, for his illustrations when publishing *De Humani Corporis Fabrica* in 1543 ( Calkins, Franciosi and Kolesari, 1999).

Could this unique association between anatomy and art be used in a contemporary context? Would it be possible to use paintings of all these renowned artists in today’s anatomy education? Can Leonardo Da Vinci, Raphael, Michelangelo and other renaissance painters teach anatomy to current medical students? The aim of this study is to assess the use of art paintings as a teaching modality in surface anatomy. Students’ perceptions and performance were investigated. Differences between the two sexes were investigated, in order to examine if art is perceived differently from male and female students. The hypothesis of this study is that the use of art paintings in learning surface anatomy does not affect students’ performance in the examinations and that it is interesting and motivating according to students’ perceptions, with no differences between the two sexes.

## Materials and Methods

### Participants and level of education

The target group of this study was the fourth-semester, undergraduate medical students of the University of Thessaly. Surface anatomy is one of the 52 student selected components (SSC), provided by the university throughout the medical curriculum and one of the 7 elective SSC provided in the fourth semester. Students were informed about the possibility of using art paintings during teaching of surface anatomy before choosing their preferred SSC. Successful completion of all previous anatomy modules (covering the entirety of anatomy and including dissection sessions) was a prerequisite for selecting the surface anatomy SCC.

### Ethical Approval

Ethical approval for the present study was obtained by the Institutional Review Board of the Univeristy of Thessaly.

### Lectures, student allocation and schedule

Students selecting the surface anatomy SSC are taught with 16, two-hour, lectures. For the needs of the present study, during the last three lectures (revision lectures), students were randomly divided into two groups. By using the auto-draw, computer program, RandomPicker (United Interactive, s.r.o., Prague, Czech Republic) two groups were created; the “Art Group” and the “Control Group”. The art group was taught surface anatomy with six hours of lectures incorporating art paintings. The control group was taught surface anatomy using classic anatomical images. The intervention was used only during revision lectures, since as an innovative idea, it should be carefully introduced and tested, without risking the components’ quality.

All lectures were prepared in PowerPoint. Slides containing art paintings were created by a Ph.D. student and the tutor using the free, Google Arts & Culture app (© Google, Inc.) to search for appropriate paintings and relevant information (artist, year, museum/gallery where the painting is exhibited). The Renaissance and the Baroque period and their contemporary artists were mostly used, since depiction of anatomical details in such paintings is usually clear and accurate. Paintings from the local art gallery were also chosen and used, using the same criteria. Examples of slides used during lectures in both groups are presented in *Appendix 1.*

### Students’ Assessment

All students were assessed by tag questions, asking them to identify surface structures in projected images. Both classical anatomical images and art paintings were used. More specifically, sixty questions used classical anatomical images and thirty questions used art paintings. All questions addressed the lowest level of cognitive objectives in bloom’s taxonomy (Level 1 or 2). Examples of questions are presented in *Appendix 2.*

### Questionnaire and Evaluation Process

After the examination, students of the art group were asked to fill in a questionnaire, in order to evaluate the learning sessions and the method that was used. The questionnaire was developed by a Ph.D. student and the tutor of the SSC. Anonymity was ensured to encourage unbiased answers. The questionnaire consisted of 16 questions, separated in four sections. It incorporated both closed-answer and open-answer questions. Closed-answer questions were yes/no questions or five-point likert-scale questions (e.g. strongly agree, agree, indifferent, disagree, strongly disagree). The first section consisted of two questions, used to collect demographical data. The second section contained questions concerning previous experience of participants with art. The third section consisted of questions evaluating the utility of art paintings in teaching anatomy. The fourth and last section contained three open-ended questions asking students to state what they liked and what they would like to be different or to be added in the educational process. The questionnaire used is provided in *Table 1.*

**Table 1.** The questionnaire completed by students of the Art group, during the evaluation process.

	Question	Type of question	Part
**1**	Sex	Multiple choice	1
**2**	Age	Open-ended	1
**3**	Do you draw / sketch / paint?	Yes/No	2
**4**	I like visiting art galleries.	Likert scale	2
**5**	Rate your knowledge in art	Likert scale	2
**6**	Do you think that the use of art paintings makes lectures more interesting?	Yes/No	3
**7**	Do you think that the use of art paintings helps you better and easier understand surface anatomy?	Yes/No	3
**8**	Do you think that presenting a brief description for each painting (painter, movement, museum) is boring?	Yes/No	3
**9**	Do you think that presenting a brief description for each painting (painter, movement, museum) is a waste of time?	Yes/No	3
**10**	Do you think that using art paintings is superior to using classical images in learning surface anatomy?	Yes/No	3
**11**	Would you like us to permanently incorporate art paintings in the surface anatomy SCC?	Yes/No	3
**12**	Do you think that the use of art paintings during lectures will help you succeed in the examinations?	Yes/No	3
**13**	Rate the contribution of lectures in your art knowledge.	Likert scale	3
**14**	What did you specifically like in the educational process?	Open-ended	4
**15**	What would you like to be different in the educational process?	Open-ended	4
**16**	What would you like to be added in the educational process?	Open-ended	4

### Statistical Analysis

Descriptive and inferential statistics was performed. Differences between students’ performance were compared using independent two sample t-test. All data were analyzed using SPSS statistical package, version 21.0 for Windows (IBM Corp., Armonk, NY). Level of statistical significance was set at p = 0.05.

## Results

### Demographics and allocation

The SSC of Surface anatomy was selected by 45 students (50% of the fourth-semester students). After random allocation in two groups (ratio 2:1), the art group consisted of 30 students (mean age=20.1, 67% ♀; 33% ♂) and the control group consisted of 15 students (mean age=19.1, 46% ♀; 54% ♂). Ratio 2:1 was used to increase the number of students using the intervention modality.

### Evaluation of the teaching method

**Figure 1.**
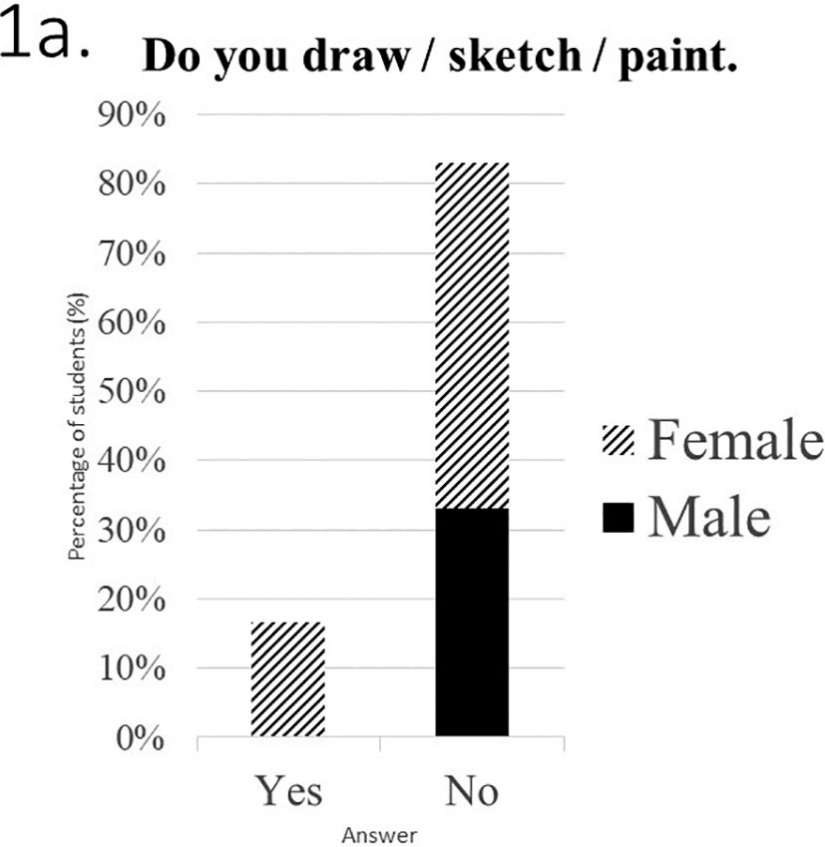

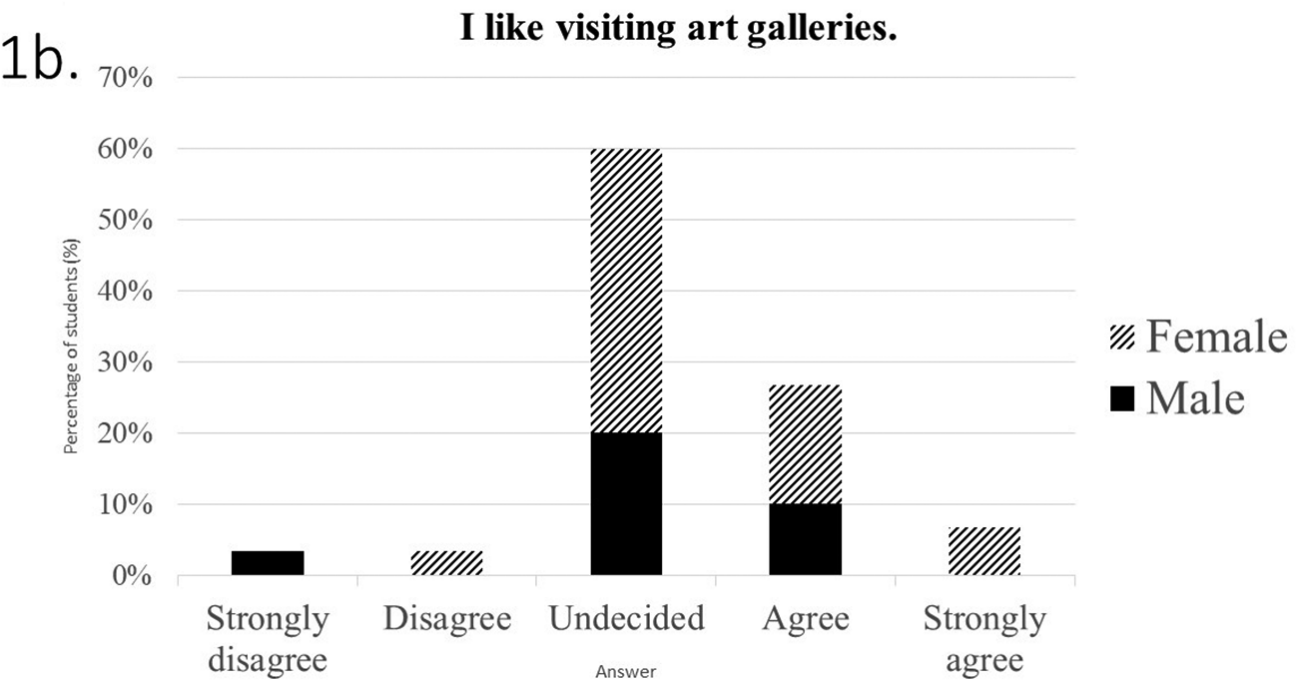

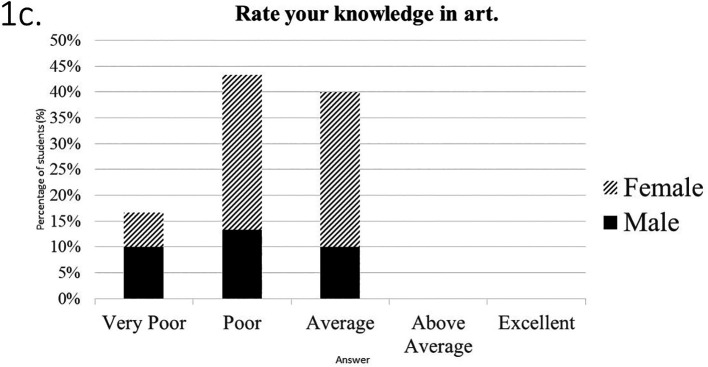
Students’ answers in the second part of the questionnaire, investigating participants’ previous experience with art. **a.** Distribution of students’ answers by sex in question 3. **b.** Distribution of students’ answers by sex in question 4. **c.** Distribution of students’ answers by sex in question 5.

Students of the art group were asked to fill in a questionnaire providing feedback from their experience during the SSC educational process. Students’ answers in the second part of the questionnaire investigating participants’ previous experience with art are presented in *Figure 1.* The majority of students didn’t paint / draw (n=25, 83%) ( Fig. 1a) and had a moderate, previous experience with art. Mean score in question 4, investigating students’ perceptions for visiting art galleries, was 3.3±0.79 with no difference between males and females (p=0.39). Mean score in question 5, asking students to rate their knowledge in art was 2.23±0.72 with no difference between male and female students (p=0.27). Figure 1b and figure 1c present distribution of students’ answers by sex in questions 4 and 5.

**Figure 2.**
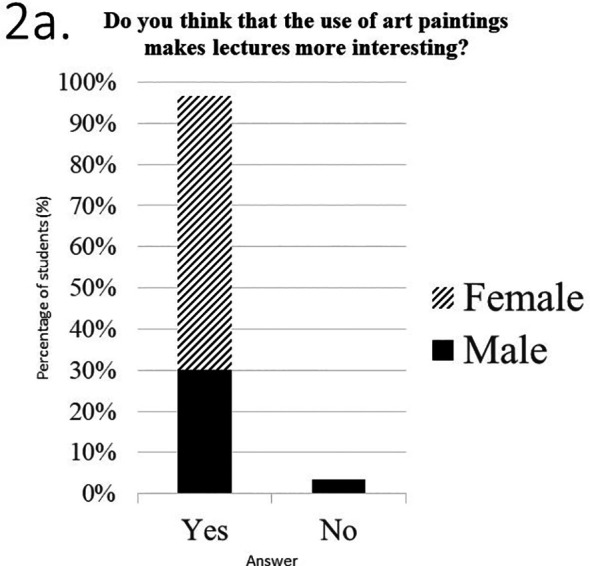

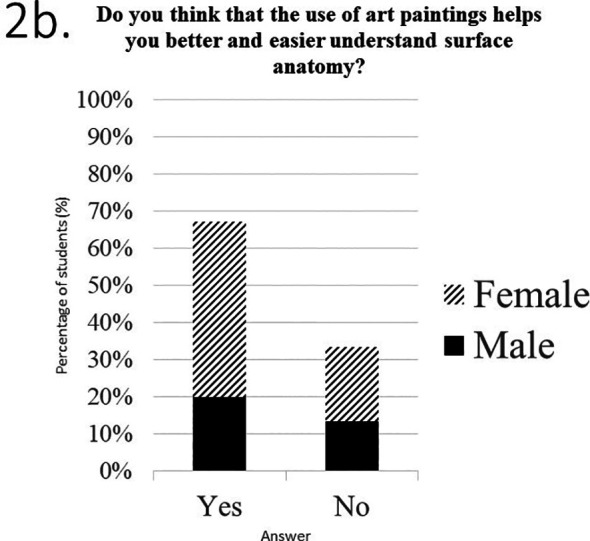

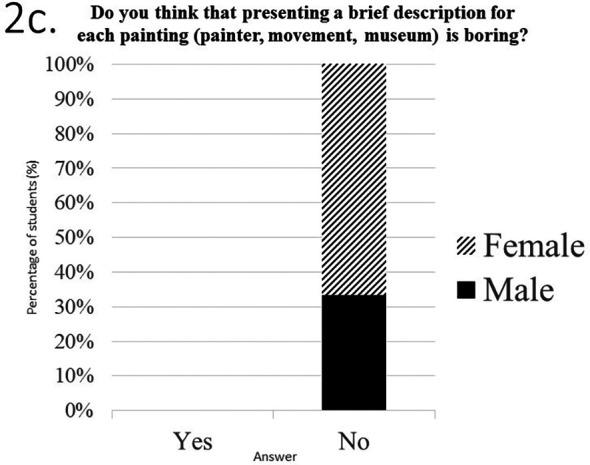

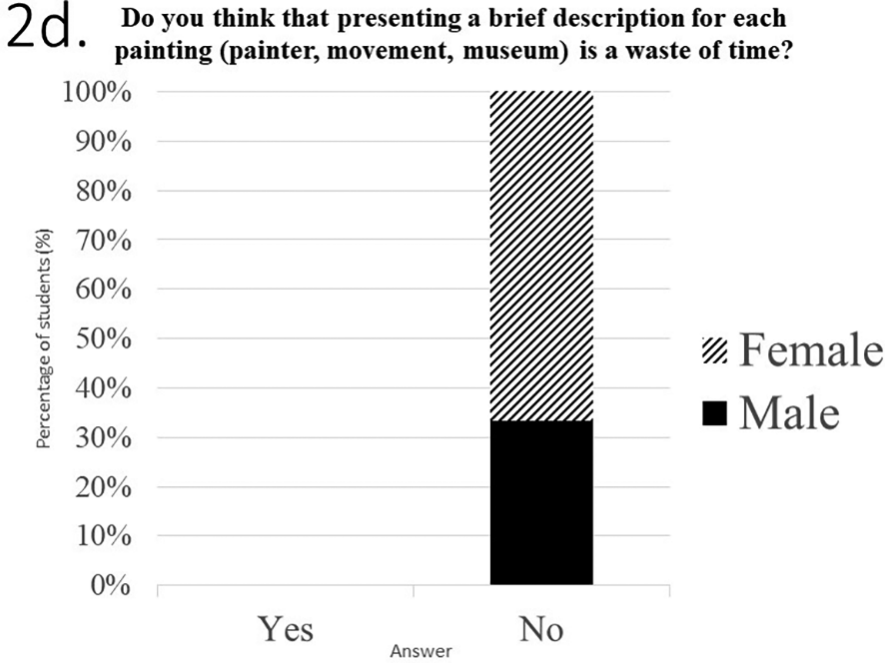

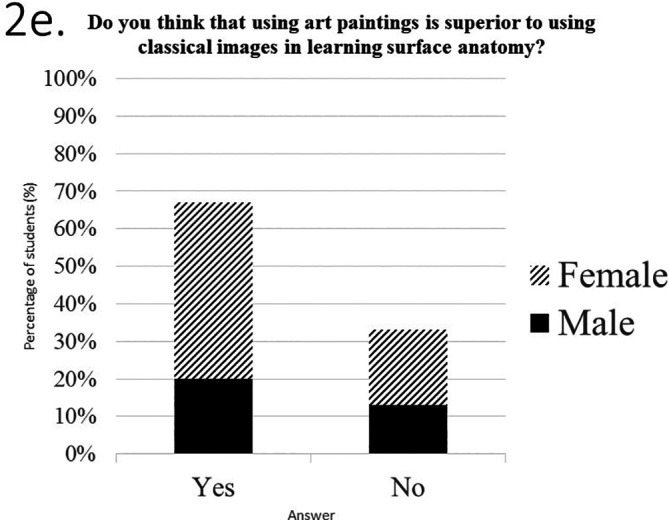

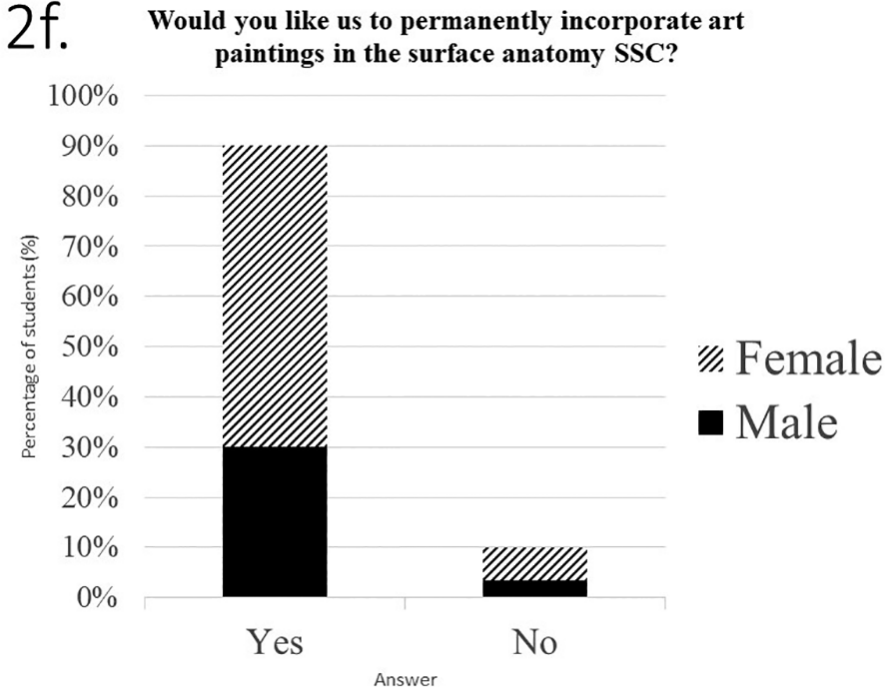

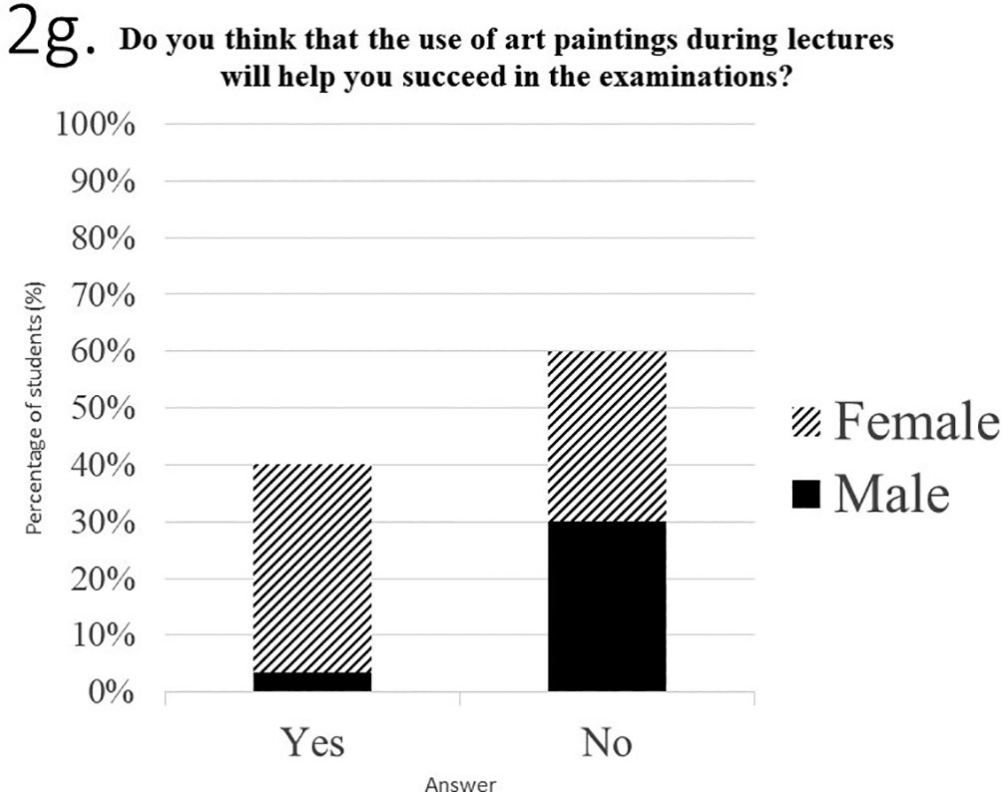

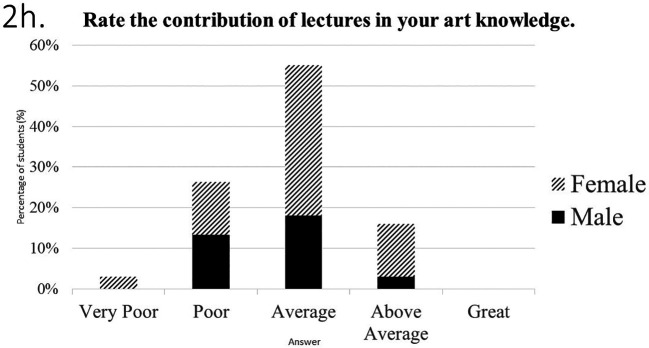
Students’ answers in the third part of the questionnaire, which asked them to evaluate the use of art paintings in teaching and learning surface anatomy. **a.** Distribution of students’ answers by sex in question 6. **b.** Distribution of students’ answers by sex in question 7. **c.** Distribution of students’ answers by sex in question 8. Distribution of students’ answers by sex in question 9. e. Distribution of students’ answers by sex in question 10. f. Distribution of students’ answers by sex in question 11. **g.** Distribution of students’ answers by sex in question 12. **h.** Distribution of students’ answers by sex in question 13.

In the third part of the questionnaire students were asked to evaluate the use of art paintings during surface anatomy teaching. Students’ answers are presented in *Figure 2.* The vast majority of students stated that the use of art paintings made lectures more interesting ( Fig. 2a) and helped them better and easier understand surface anatomy ( Fig. 2b). All students also said that a brief description of the painting (painter, movement, museum where the painting is exhibited) was neither boring, nor a waste of time ( Fig. 2c, 2d). Moreover, 67% of all students (n=20) stated that they consider the use of art paintings superior to classical images in learning surface anatomy ( Fig. 2e), confirming the initial hypothesis of this study, and 90% (n=27) would like such paintings to be permanently incorporated in lectures of surface anatomy ( Fig. 2f). However, 60% (n=18) answered that they don’t believe this modality would help them succeed in the examinations ( Fig. 2g). Finally, 70% (n=21) believe that this teaching modality had at least an average contribution to their knowledge in art ( Fig. 2h).

In the fourth and last part of the questionnaire, students were asked to state what they liked and what they would like to be different or to be added in the educational process. In these open-ended questions students cited the benefits of using art paintings in a way reflecting the previously presented statistical data. Specific students’ comments included: “interesting and creative approach in learning surface anatomy”, “I enjoyed learning art and anatomy together” and “it surely helped with my anatomy revision”. In the question “What would you like to be different?” comments included: “use art paintings in all sessions” (n=21, 70%), “6 hours of art lectures are not enough” (n=24, 80%), “use classic images and art paintings side by side” (n=12, 40%), “use the famous comic hero Hulk for muscle surface anatomy” (n=4, 14%). Finally, in the question “What would you like to be added?” students answered that a visit to museum for a live anatomy lesson using paintings and sculptures would be an interesting idea, as a plan for the forthcoming year (n=18, 60%).

### Students’ performance

**Figure 3.**
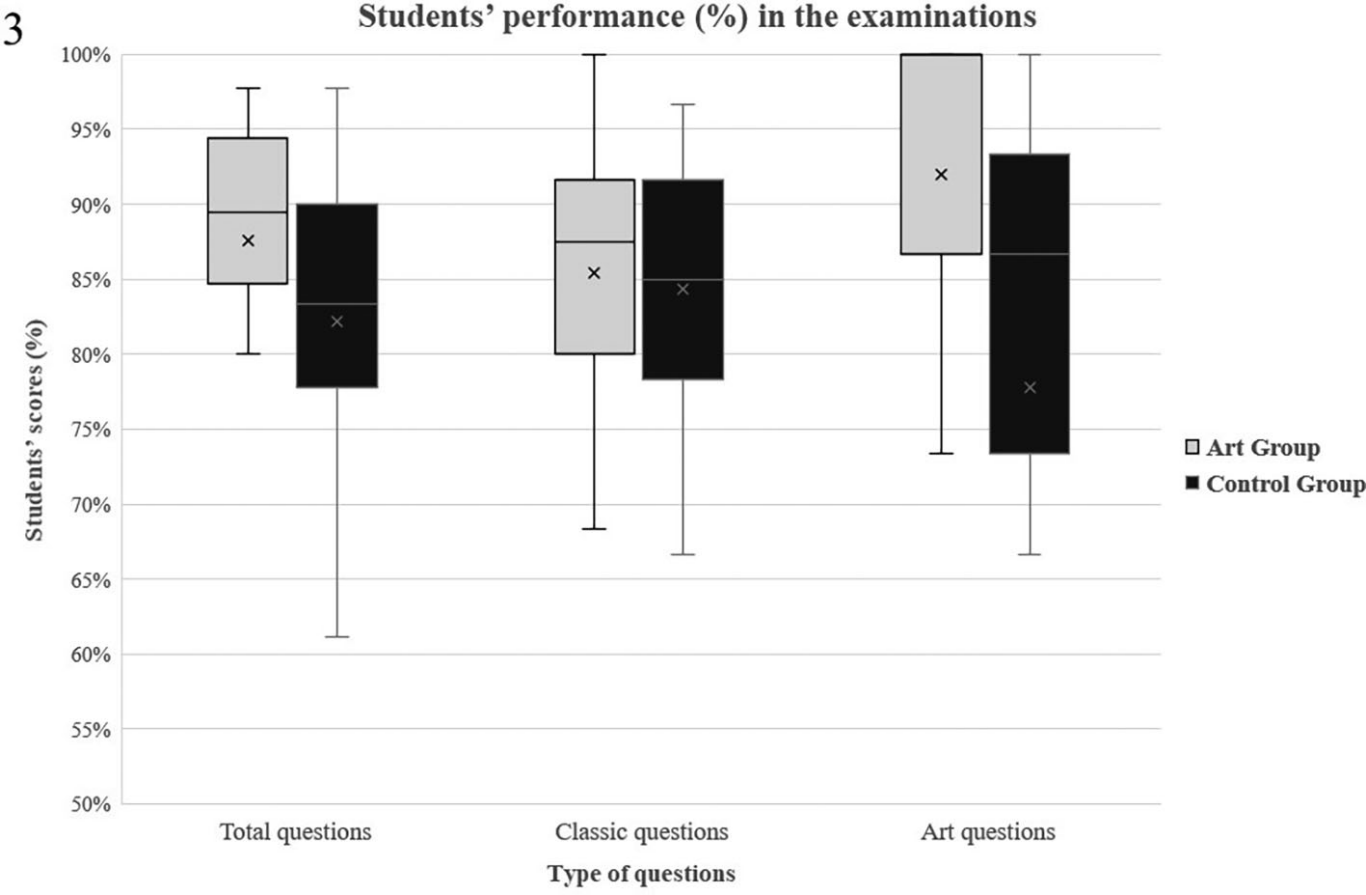
A boxplot of students’ scores (%) in the examinations, with whiskers from minimum to maximum. Performance of students in each group (control group and art group) is depicted for each question type (total, art and classic questions). Segment inside the boxplot shows the median and “x” shows the mean.

Both groups were assessed by 90 tag questions (60 using classic anatomical images and 30 using art paintings), asking them to identify anatomical structures. In questions using classic anatomical images, there was no significant difference between the two groups (Control group: mean=50.6±4.9; Art group: mean=51.2±5.2, p=0.69). In questions using art paintings, patients of the art group performed significantly better (Control group: mean=23.3±6.4; Art group: mean=27.6±4.3, p=0.01). In total questions no significant difference between the two groups was observed, comfirming the initial hypothesis of this study (Control group: mean=73.9±9.4; Art group: mean=78.8±8.6, p=0.09). Results are presented in *Figure 3.* No significant difference between men and women was observed regarding total (p=0.60), classic (p=0.70) or art questions (p=0.13) in the art group. In the control group, no difference was observed in total questions (p=0.23) and classic questions (p=0.67), but female students performed better in art questions (♂: 20±7.1; ♀:27.1±2.5, p=0.02).

## Discussion

### Students’ perceptions regarding use of paintings as a teaching modality

Surface anatomy is an integral part of medicine, since it enables students to improve their skills in clinical examination, interventional procedures and interpretation of diagnosing images ( Leonard *et al.*, 1999). An increase in research addressing teaching and learning in surface anatomy has been observed the last few years ( Azer, 2013). However, few teaching methods, incorporating art in teaching surface anatomy, have been reported so far. According to recent literature, another popular method, highly appreciated by students in learning surface anatomy, is body painting ( McMenamin, 2008; Finn and McLachlan, 2010) which is considered as a fun and cheap teaching modality ( Finn, 2010). In this study, the vast majority of students stated that the use of art paintings made lectures more interesting. Almost 70% (n=20) stated that art paintings helped them better and easier understand surface anatomy and the same percentage considered them superior to classic anatomical images. Finally, 90% (n=27) stated that art paintings should be permanently incorporated in the current curriculum, supporting our viewpoint that art paintings are a promising teaching modality which should be furtherly tested.

Other teaching and learning methods, like peer physical examination and palpation in living anatomy classes have been also proposed, providing an additional dimension in the teaching process ( Collett *et al*., 2009; Chinnah *et al*., 2011). Imaging studies have been successfully used as well in teaching surface anatomy, usually in conjunction with traditional methods. Ultrasound was perceived as an innovative and effective way to learn surface anatomy by medical and dental students and can be also performed in students - volunteers ( Ivanusic *et al*., 2010; Stringer *et al*., 2012; Swamy and Searle, 2012). Full-body digital x-ray images were also considered beneficial combined with drawing, especially for anatomy of the thorax ( Kotze, Mole and Greyling, 2012). And even yoga and pilates, which are considered to promote enjoyment, physical and mental health, have been used in teaching surface anatomy ( McCulloch *et al.*, 2010).

### Impact on performance

Based on their six-hour experience, 60% (n=18) of students stated that the use of art paintings won’t help them succeed the examinations. Many students also asked for more hours of “art” lectures (n=24, 80%) and a visit to the museum for a live surface anatomy lesson in paintings and sculptures (n=18, 60%). Based on scores of the examination process, performance of the two groups did not significantly differ in total (p=0.09), or in questions using classic anatomical images (p=0.69). However, students from the art group performed significantly better in questions using art paintings (p=0.01), which seems reasonable since they were familiar with such pictures. Overall, the use of art paintings did not improve students’ scores, but nevertheless 6 hours of revision lectures are not enough to improve students’ performance, which is also shown by the need for more lectures and a visit to a museum, addressed by the majority of students. So far, only one randomized control study examining impact in performance was identified in the literature. Azer compared reading text and drawing abdominal organs with reading text and answering short-answer questions, concluding that both methods improve students’ scores but learning by drawing is superior ( Azer, 2011).

### Art in medical education

Various uses of art in medical education have been explored the last few years. Improving observational skills in medical students is one of them ( Boisaubin and Winkler, 2000; Shapiro, Rucker and Beck, 2006). Visits to museums and examination of painted portraits, sculptures and other artworks ( Bardes *et al*., 2001; Elder *et al*., 2006; Naghshineh *et al*., 2008; Schaff *et al*., 2011; Friedlaender and Friedlaender, 2013) have shown to improve students’ skills in observing patients and recognizing signs and symptoms. Furthermore, targeting at understanding and empathy through engaging with Frida Kahlo’s paintings ( Darbyshire, 1994) or by creating artwork based on lives of patients with chronic illness ( Kumagai, 2012), has been reported in the literature. Art has been also used to assist anatomy learning. An “analyzing art” course aiming at developing participants’ observational skills in anatomy, through studying pre-existing paintings and photographs, was recently reported ( Bell and Evans, 2014). In this study, students strongly believed that art can play a role in medical education and that skills used through art can be beneficial in their future practice as doctors ( Bell and Evans, 2014). Moore *et al*. also tried to integrate humanities and develop observational skills by incorporating drawing of plastinated specimens and using lectures in contemporary artists in an art and anatomy workshop ( Moore *et al.*, 2011). Students felt that this approach gives a new perspective to the human body, which is a worthwhile investment in anatomy learning.

However, improving students’ observational skills was not the goal of the present study, which, to our knowledge, is the first to describe the use of art paintings in teaching surface anatomy. In this study, use of art was also welcomed by students. Although there were only few students painting or drawing (n=5, 18%) with poor or average knowledge in art and with a moderate desire for visiting art galleries, there were no participants stating that the painting description (painter, museum, movement etc.) was boring or a waste of time. In fact, students felt that the use of this teaching modality had also an average contribution in their knowledge in art, which can be only perceived as a positive fact in the context of pedagogical teaching.

At this point, it is also worth mentioning that female students of the control group performed better in art questions compared to their male classmates (p=0.02). This may be a clue that female students have a closer relationship with art, which helped them score higher in the examinations, although they had not been taught with lectures comprising art paintings. This observation could be tested in future studies with larger samples.

### Limitations

The small sample size could be considered as a limitation. Additionally, lectures using art paintings were revision lectures, lasting only 6 hours. Both groups used classic anatomical images during the rest of the semester. However, considering that this is the first try to use art paintings in teaching and learning surface anatomy, this study could be used as a guide for future studies.

### Conclusions

Students enrolled in this SSC perceived the use of art paintings as a useful learning method which made lectures more interesting and improved understanding. Although no improvement in examination scores was observed, students would like to use again art paintings, since no one found the lectures’ content boring and an average contribution in their art knowledge was also achieved. Since there is literature to support use of art in medical education and research in this area is at an early stage, future studies should be performed testing the true potential of art paintings in surface anatomy. Randomized samples and other evidence-based methods should be tested to investigate if art can benefit medical students in surface anatomy learning and whether it should be used alone or in combination with other teaching methods in future curricula.

## Take Home Messages

• Art paintings could be a valuable adjunct in teaching surface anatomy.
• Students perceived the use of art paintings as a useful learning method which made lectures more interesting and improved understanding.
• Although no improvement in examination scores was observed, an average contribution in students’ art knowledge was achieved.
• Future studies could test the true potential of paintings in surface anatomy.

## Notes On Contributors

**Vasileios Mitrousias** is a resident doctor in the Department of Orthopaedic Surgery, University Hospital of Larissa, Greece. He holds a PhD in anatomy education, and he teaches anatomy to physiotherapy and nursing students. His research interest is in medical education and orthopaedic surgery. ORCID iD: https://orcid.org/0000-0002-2040-6804

**Kyriaki Baxevanidou** is postgraduate student in the Department of Anatomy, School of Health Sciences, University of Thessaly and resident in the Department of General Surgery, General Hospital of Larissa, Greece. Her research interest is in medical education and breast surgery.

**Aristeidis Zibis** is Associate Professor of Anatomy in the Department of Anatomy, School of Health Sciences, University of Thessaly, Greece and orthopaedic surgeon. He teaches anatomy to medical students and residents. He is responsible of a series of workshops in clinical anatomy of the shoulder, knee and the ankle. His research interest is in medical education and orthopaedic surgery. ORCID iD: https://orcid.org/0000-0001-7122-4317

## Appendices

### Appendix 1

1. A typical slide used during teaching surface anatomy in the Art Group. The famous painting “The creation of Adam” of Michelangelo is used.

2. A typical slide used during teaching surface anatomy in the Control Group. A typical anatomical image produced in our department is used.

### Appendix 2

1. A typical “art” question used during examinations. The painting “Ignudo” of Michelangelo is used.

2. A typical “classic” question used during examinations. An anatomical image of our department is used.
