# Supplementary material for: Art & anatomy: an everlasting relationship creating new insights in teaching surface anatomy
Source: MedEdPublish (2016). 2020 Jan 28;9:23. [Version 1] doi: 10.15694/mep.2020.000023.1 (PMC10697463; doi:10.15694/mep.2020.000023.1)
Supplement: Peer review history

# Reviewer report: Reviewer response for version 1

**DOI:** 10.21956/mep.19967.r30349

**Published:** 2020-03-05

**Authors:** P Ravi Shankar

**Affiliations:** 1 American International Medical University

This review has been migrated. The reviewer awarded 4 stars out of 5 This is an interesting paper. The authors describe the use of classic paintings to revise surface anatomy to a group of medical students. Art and anatomy have had a close relationship as described by the authors. I have been using art in a medical humanities module which I facilitate to explore various aspects of human relationships with special reference to the patient-doctor relationship. The authors have described the methodology of the study well. They also found that female students had a greater interest in art which has been shown previously. Students perception about the initiative was positive. With widespread availability of online image sources, educators in developing nations should be able to use art in medical education without much difficulty. This paper will be of interest to all medical educators.

Reviewer Expertise:

NA

No decision status is available

# Reviewer report: Reviewer response for version 1

**DOI:** 10.21956/mep.19967.r30348

**Published:** 2020-02-03

**Authors:** Barbara Jennings

**Affiliations:** 1 Norwich Medical School

This review has been migrated. The reviewer awarded 4 stars out of 5 I will recommend this article to other readers for the art history discussed the introduction, which is a brief but fascinating summary of a centuries-old association between art and the study of human anatomy. The authors cite some interesting literature exploring the value of art and experiential learning in both the Introduction and Discussion of this paper.In their introduction the authors ask “Can Leonardo Da Vinci, Raphael, Michelangelo and other renaissance painters teach anatomy to current medical students?” When I read this, I wondered about the place of work by twentieth century and contemporary artists too (e.g. Lucian Freud, Jenny Saville, Marc Quinn, Toyin Ojih Odutola, etc.) exploring art created with a more diverse range of subjects may be educationally useful too; with regard to both anatomy-skills and fundamental values. The authors describe a thorough evaluation of an educational initiative to include the use of art in surface anatomy lectures during a taught student-selected component. The evaluation explores student perspectives in the intervention and its influence on their performance in the anatomy examination questions.The organisation, governance and approach taken were clearly described by the authors in the Methods section.Summary statistics and a narrative summary were presented in the results. These were to evaluate the student’s perception of the initiative and to identify areas for curriculum development.There are quantitative aspects to the evaluation of student performance. And the authors do cite sample size as one of the study limitations. However, I think that authors could present a power calculation for the questions asked in a revised version, and in this case the authors could link this to their discussion of “impact on performance” in the discussion. To draw conclusions about the measurable impact of an intervention it is important to consider the effect size expected for that intervention - and to determine the number of participants needed to include. A small study like this may be under-powered so that a small positive effect could be missed (overlooked). I understand that the number of participants here would be constrained by SSC numbers, but the risk of a type 2 error should be acknowledged in the discussion of study limitations. Overall, I really enjoyed reading about this well conducted study, with its clear and interesting aims.

Reviewer Expertise:

NA

No decision status is available

# Reviewer report: Reviewer response for version 1

**DOI:** 10.21956/mep.19967.r30350

**Published:** 2020-01-29

**Authors:** Felix Silwimba

**Affiliations:** 1 University of Lusaka

This review has been migrated. The reviewer awarded 5 stars out of 5 this is a good study . the use of art in medical education. i think it is not only limited to surface anatomy. educators in LIMC countries can extend further to other medical disciplines. I learnt innovation in this article

Reviewer Expertise:

NA

No decision status is available
